# BURNOUT AND FATIGUE AND THE EMPLOYMENT OF NURSES IN SEVERAL WORKPLACES: A CROSS-SECTIONAL STUDY

**DOI:** 10.13075/ijomeh.1896.02613

**Published:** 2025

**Authors:** Klaudia Dubis, Angelika Korzeń, Zuzanna Sroka, Katarzyna Wójcik, Łukasz Lompart, Marlena Padykuła, Sylwia Kocur, Urszula Kalemba, Iwona Malinowska-Lipień

**Affiliations:** 1 Jagiellonian University – Medical College, Institute of Nursing and Midwifery, Faculty of Health Sciences, Kraków, Poland; 2 University Children's Hospital in Kraków, Kraków, Poland; 3 Specialist Hospital Ludwik Rydygier in Kraków, Long-Term Care Facility, Kraków, Poland

**Keywords:** fatigue, burnout, nursing, additional employment, multiworkers, monoworkers

## Abstract

**Objectives:**

The purpose of this study is to assess the relationship between burnout and fatigue and the employment of nurses in several workplaces.

**Material and Methods:**

The study group consisted of 234 professionally active female and male nurses employed in medical facilities in various regions of Poland. The study was conducted using an online survey consisting of 2 parts: the *Maslach Burnout Inventory* (MBI) questionnaire and an original questionnaire including questions about demographic, professional and fatigue data.

**Results:**

More than half of the nurses (54.70%) worked in an additional place of work. Among those who took on additional work, as many as 87.50% (112 people) reported feeling tired in the last 6 months and 50% (64 people) in this group noticed a negative impact of additional work on their mental health. The analysis also showed a statistically significant relationship (p = 0.038) between employment in an additional place and the occurrence of headache in employees.

**Conclusions:**

Financial factors are the main reason for nurses taking up additional employment. Analysis of the results showed that working in >1 place significantly increases the level of fatigue compared to employment in 1 facility. Nurses employed in >1 full-time position were characterized by a higher level of burnout and increased emotional exhaustion.

## Highlights

- Extra jobs are worked by 54.7% of nurses; 82.8% cite financial need as the main reason.
- Chronic fatique is reported by 87.5%; 50% note worsened mental health due to extra work.
- Multiple-job nurses are older, better educated, and more professionally trained.
- Extra jobs would be quit by 78.9% if primary pay were sufficient – pointing to a system issue.

## INTRODUCTION

According to World Health Organization (WHO), burnout is a clinical condition resulting from chronic stress related to work, manifested by “a feeling of exhaustion or depletion of energy; increased psychological distance from work or a sense of negativity, cynicism related to the work performed and reduced professional effectiveness” [1]. Burnout syndrome occurs when an employee begins to feel overburdened with duties, unwillingness to develop professionally and lack of satisfaction with the work performed. According to Maslach [2,3], the main elements of burnout are:

– depersonalization, i.e., in relations to medical personnel, loss of perception of the patient, a person who has emotions, problems and treating him as another case to be cured;
– emotional exhaustion, i.e., lack of desire, energy to perform the job, which is manifested by a feeling of fatigue and the occurrence of somatic symptoms such as headache or insomnia;
– a decrease in the sense of one's own achievements, i.e., treating oneself as a person incapable of achieving anything – this is the most advanced stage of burnout [2,3].

The burnout process is long-lasting and imperceptible at its initial stage. Over time, the person becomes less and less diligent in their duties, makes more mistakes, shows reluctance to work and increases psychophysical tension, which results in emotional exhaustion. Somatic symptoms may also appear. It may also lead to reaching for stimulants such as alcohol, medication, or even suicide attempts [2,4]. The stages of burnout are successively enthusiasm, stagnation, frustration, and apathy [5]. Nursing staff should pay special attention to factors that may contribute to the development of burnout, such as specific working conditions (shift work, direct contact with people), psychological burden (suffering and death of the patient), insufficient number of nursing staff, difficult atmosphere at work, or low professional prestige [6].

Fatigue among nursing staff is a very common phenomenon that reduces the quality of care and efficiency. Long-term fatigue leads to deterioration of health and reduces the productivity of employees [7,8]. Fatigue is a state of mental or physical exhaustion, which is characterized by reduced work capacity [7]. The causes that contribute to the occurrence of fatigue include insufficient rest, excessive effort, routine, experiencing strong emotions and burnout. Chronic fatigue syndrome is also distinguished, which is a disease resulting from an unjustified feeling of fatigue lasting >6 months with accompanying somatic symptoms. Shift work can contribute to chronic fatigue, which manifests itself as apathy, difficulty sleeping or exhaustion [9].

Double professional practice in nursing is widespread and enables healthcare workers to stay in their workplace, as they take on additional work in private facilities without having to leave the public sector, which is their primary place of work [10,11]. Shift work and 12-hour shifts provide an opportunity for additional employment, which enables a smooth transition from one job to another and unfortunately involves less time for rest and sleep [12]. Additional job is undertaken by nursing staff for various reasons, especially due to additional remuneration or gaining new experience and knowledge [10].

The study may show significant results of the impact of taking up employment in several places of work by nursing staff on the occurrence of the phenomenon of occupational burnout. The results may be used to reflect on the significance of working in several places or the amount of working time in the second employment.

### Objective of the study

Assessment of the relationship between burnout and fatigue and the employment of nurses in several work-places.

## MATERIAL AND METHODS

A cross-sectional study was conducted in February–May 2024 using an online survey distributed via the Internet. The survey was directed exclusively to a group of nurses using closed groups on social media platforms. Respondents were informed about the voluntary nature of participation, the anonymity of the study, and the possibility of withdrawal at any stage.

The research group consisted of female and male nurses employed in various regions of Poland. The final analysis included 234 people who met the inclusion criteria. The sample was random. The inclusion criterion included active practice of the nursing profession and consent to participate in the study. The exclusion criteria included the lack of consent to participate in the study and the lack of active professional practice (i.e., being on maternity leave, inability to work due to long-term illness, retirement).

The study used a questionnaire consisting of 2 parts. The first was the *Maslach Burnout Inventory* (MBI) questionnaire, a standardized tool for assessing burnout. This tool consists of 22 items relating to key aspects of burnout: emotional exhaustion (EE), depersonalization (DE) and reduced sense of personal accomplishment (PA). The maximum scores that can be obtained in the individual subscales are: 54 for EE, 48 for PA and 30 for DE. Separate results are obtained for each of the 3 subscales, but it is possible to determine the general level of burnout. The higher the value in EE and DE subscales and the lower the PA value, the higher the level of burnout. The EE scale includes 9 questions [2,4,13,14], the lack of PA includes 8 questions [15,16], and the DE scale includes 5 questions [3,5,6,17]. The items in the EE and DE subscales are negative, while the items in the PA subscale are positive. Respondents assessed each statement on a scale 0–6, where 0 meant that they had never experienced such feelings, while 6 indicated that they experienced these feelings every day. The results for individual subscales were calculated separately, in accordance with methodological recommendations [16].

The second part of the survey was an original questionnaire designed by Iwona Malinowska-Lipień and used for the purposes of the study [12]. The survey included a total of 34 questions, including questions about sociodemographic data regarding gender, age, family situation, place of residence, education, employment status, as well as seniority and place of work. The remaining questions concerned the issue of additional professional practice. Additionally, this part contained 10 questions regarding the subjective assessment of physical and mental well-being over the last 6 months. The questions were closed-ended, with response options: “yes,” “no,” or “I don't know.”

The study was conducted in accordance with the assumptions of the Declaration of Helsinki. The consent of the Bioethics Committee of the Jagiellonian University – Collegium Medicum, Kraków, Poland, No. 118.6120.223.2023.

The statistical analysis was performed using Statistica 9.1 software (StatSoft, Kraków, Poland). The values of the analysed quantitative variables were presented using the mean, median, lower and upper quartile, min and max. values and standard deviation, and qualitative variables using the frequency and percentage. The relationship of qualitative variables was assessed using the χ^2^ test (in the case of 2×2 tables and small numbers, the Yates correction was used). The normality of the distribution of variables in the studied groups was checked using the Shapiro-Wilk normality test. The Student's t-test was used to examine differences between 2 groups, and in the case of failure to meet the conditions for its application, the Mann-Whitney test. The assessment of differences between ≥3 groups was performed using ANOVA analysis of variance (together with Tukey's RIR *post hoc* test), and in the case of failure to meet the conditions for its application, the Kruskal-Wallis test. The significance level of p < 0.05 was adopted, indicating the existence of statistically significant differences or dependencies.

## RESULTS

The article employs the concept of „multiwork”, referring to individuals working >1 job as „multiworkers”, and those with only 1 job as „monoworkers” [18]. Table 1 presents the characteristics of the study group, distinguishing between multiworkers and monoworkers.

**Table 1:** Sociodemographic and occupational characteristics of nurses employed in medical facilities, with additional and 1 job, Poland, February–May 2024

Variable	Participants (N = 234)			
	with additional job (N = 128, 54.70%)		with 1 job (N = 106, 45.30%)	
	n	%	n	%
Gender				
women	110	85.94	103	97.17
men	18	14.06	3	2.83
Age				
<25 years	15	11.72	54	50.94
25–44 years	68	53.13	36	33.96
≥45 years	45	35.16	16	15.09
Education				
secondary medical	6	4.69	5	4.72
bachelor's degree	63	49.22	92	86.79
master's degree	59	46.09	9	8.49
Work experience				
<1.5 years	9	7.03	48	45.28
1.5–5 years	30	23.44	32	30.19
5–20 years	45	35.16	11	10.38
>20 years	44	34.38	15	14.15
Place of work				
hospital	117	91.41	100	94.34
other (clinic, other)	11	8.59	6	5.66
Employment sector				
private	9	7.03	14	13.21
state	119	92.97	92	86.79
Employment dimension				
full time	117	91.41	96	90.57
part time	3	2.34	4	3.77
contracts of mandate	4	3.13	4	3.77
contracts	4	3.13	2	1.89

The majority of respondents were employed in medical facilities, with smaller groups working in nursing homes or as academic teachers. The most common additional workplaces included clinics, hospital departments, other healthcare settings, and universities. Employment was divided between the public and private sectors. In their second job, most respondents worked under a contract for services, with fewer employed part-time, under civil contracts, or full-time. Working hours varied monthly, and the duration of additional employment most often ranged from several months to a few years. Financial reasons were the main motivation for taking up a second job. Most participants indicated they would quit if their primary job provided adequate income, while some expressed willingness to continue regardless of financial circumstances (Table 2).

**Table 2:** Characteristics of the studied group of nurses working in an additional workplace in terms of profession, Poland, February–May 2024

Variable	Participants (N = 128)	
	n	%
Nature of the position in the additional workplace		
nurse	112	87.50
university teacher	7	5.47
other	9	7.03
Additional workplace		
hospital department	41	32.03
clinic	49	38.28
university	7	5.47
other	31	24.22
Sector of additional workplace		
private	58	45.31
state	70	54.69
Type of employment in the additional place of work		
full-time	10	7.81
part-time	16	12.50
contract of mandate	90	70.31
contract	12	9.38
Time worked in the additional place of work		
<10 h/month	4	3.12
10–40 h/month	40	31.25
40–80 h/month	44	34.38
80–160 h/month	36	28.13
>160 h/month	4	3.12
Length of service in the additional place of work		
<month	1	0.78
1–6 months	18	14.06
6 months – 1 year	25	19.53
1–3 years	32	25.00
3–5 years	20	15.63
5–10 years	21	16.41
10–15 years	7	5.47
>15 years	4	3.12
Main reason for taking up a second job		
personal reasons	10	7.81
financial reasons	106	82.81
willingness to use free time	7	5.47
other	5	3.91
Willingness to give up additional job if main job provides financial satisfaction		
yes	101	78.91
no	27	21.09

Table 3 presents the responses to selected questions from the original questionnaire concerning the subjective assessment of physical and mental well-being over the past 6 months. The statistical analysis showed statistically significant differences in perceived fatigue and employment in an additional job (p = 0.034), which means that additional work may have a greater impact on the level of fatigue compared to working in 1 place. The analysis also showed a statistically significant relationship (p = 0.038) between employment in an additional place and the occurrence of headache in employees.

**Table 3:** Responses to selected questions from the original questionnaire concerning the subjective assessment of physical and mental well-being over the past 6 months in nurses with additional and 1 job, Poland, February–May 2024

Variable	Participants (N = 234)				p
	with additional job (N = 128)		with 1 job (N = 106)		
	n	%	n	%	
Frequent headaches in the last 6 months					0.038
yes	88	68.75	56	52.83	
no	38	29.69	46	43.40	
I don't know	2	1.56	4	3.77	
Frequent sore throat or hoarseness in the past 6 months					0.578
yes	39	30.47	37	34.91	
no	86	67.19	68	64.15	
I don't know	3	2.34	1	0.94	
Occurrence of enlarged and painful cervical or axillary lymph nodes in the last 6 months					0.694
yes	19	14.84	13	12.26	
no	107	83.59	90	84.91	
I don't know	2	1.56	3	2.83	
Frequent feeling of fatigue in the last 6 months					0.034
yes	112	87.50	79	74.53	
no	14	10.94	22	20.75	
I don't know	2	1.56	5	4.72	
Frequent feeling of muscle pain in the last 6 months					0.320
yes	69	53.91	47	44.34	
no	56	43.75	55	51.89	
I don't know	3	2.34	4	3.77	
Frequent feeling of joint pain in the last 6 months					0.075
yes	64	50.00	39	36.79	
no	63	49.22	67	63.21	
I don't know	1	0.78	0	0.00	
Feeling of unwell after exertion for >24 h in the last 6 months					0.082
yes	67	52.34	40	37.74	
no	55	42.97	59	55.66	
I don't know	6	4.69	7	6.60	
Feeling of deterioration in memory and concentration in the last 6 months					0.214
yes	90	70.31	63	59.43	
no	32	25.00	37	34.91	
I don't know	6	4.69	6	5.66	

The study of the effect of age on burnout showed that the total level of burnout was similar across age groups (p = 0.301). Emotional exhaustion, on the other hand, increased with age, with the lowest scores among those <25 years of age (p = 0.004). In the case of DE and PA, there were no significant differences between groups (p > 0.05). Thus, only EE differed by age, while other aspects of burnout were independent of age.

The overall burnout score among individuals working in multiple positions was 51.89, while among those working in a single position it was 50.55 (p = 0.577). Emotional exhaustion scores were slightly higher among those employed in >1 position (23.52) compared to those working in a single position (20.91) (p = 0.051). Mean DE scores were very similar between the groups (7.47 vs. 4.48) (p = 0.874). No differences were found in the sense of PA between individuals working in multiple positions and those employed in 1 position (20.90 vs. 2.16) (p = 0.236).

The study results indicate that individuals with secondary medical education experience a higher overall level of burnout compared to those with a master's degree. This difference is particularly evident in the area of DE. Emotional exhaustion also appeared to be more pronounced among those with lower educational attainment, although the difference was not statistically significant. Overall, the findings suggest that education level may influence susceptibility to burnout (Table 4).

**Table 4:** Characteristic of the main elements of burnout in group of studied nurses due to type of school graduated, Poland, February–May 2024

Burnout	M	SD	Me	Q1	Q3	Min.	Max	p
Total score of burnout								0.048
secodary medical education	60.45	15.35	63.00	57.00	70.00	18.00	74.00	
bachelor's degree	51.21	18.26	53.00	42.00	62.00	7.00	100.00	
master's studies	49.97	18.62	48.50	37.50	62.50	6.00	98.00	
Emotional exhaustion								0.129
secondary medical education	26.64	9.88	26.00	18.00	34.00	9.00	43.00	
bachelor's degree	21.55	11.27	21.00	13.00	29.00	0.00	53.00	
master's studies	23.44	10.61	22.00	16.00	30.50	1.00	52.00	
Depersonalization								0.013
secondary medical education	11.09	6.02	12.00	5.00	18.00	4.00	19.00	
bachelor's degree	7.75	5.79	7.00	3.00	11.00	0.00	28.00	
master's studies	6.26	5.90	4.50	2.00	8.50	0.00	24.00	
Personal accomplishment								0.481
secondary medical education	25.27	10.52	25.00	18.00	34.00	11.00	46.00	
bachelor's degree	26.09	9.93	26.00	19.00	33.00	1.00	47.00	
master's studies	27.74	9.31	27.00	21.00	34.50	4.00	47.00	

Secondary medical education N = 11, bachelor's degree N = 155, master's studies N = 68.

In the study of the effect of job seniority on burnout, no significant differences were found in the overall level of burnout (p = 0.447). However, those with longer job seniority had higher levels of EE (p = 0.016) and a greater sense of personal achievement (p = 0.003) compared to those with shorter job seniority. The results suggest that longer job seniority may lead to greater EE, but also a greater sense of professional success.

The study of burnout depending on the workplace showed that people working in hospital wards and clinics have higher levels of burnout and EE than those working in universities and other places. These differences were statistically significant (p = 0.007 for general burnout and p = 0.004 for EE). No significant differences were found in terms of DE and PA. The results suggest that working in hospitals and clinics is associated with higher levels of burnout.

The analysis of burnout in relations to the occurrence of fatigue showed that people without fatigue had significantly lower burnout scores (43.32 vs. 58.22), EE (17.39 vs. 26.66) and DE (5.72 vs. 9.01) compared to people with fatigue p < 0.05. However, the differences in PA between the groups were small and insignificant. The conclusions suggest that fatigue is strongly associated with a higher level of burnout.

## DISCUSSION

Studies by Poghosyan et al. [19] indicate that professional burnout significantly reduces the quality of nursing care, and people affected by this phenomenon are less likely to provide high-quality services. This problem affects nurses all over the world, regardless of cultural, social or economic differences. In the face of the global shortage of nursing staff, ensuring high and safe quality of services is becoming extremely difficult, especially in the context of burnout [19]. According to the Central Register of Nurses and Midwives, there are 315 670 registered nurses in Poland, of which only about 74% work in the profession. On average, there are 62 nurses per 10 000 patients, and the average age of a nurse in Poland is >54 years [13]. These data indicate how big the problem of staff shortages among nurses is in Poland, which may translate into fatigue and, consequently, the occurrence of burnout. The authors' study showed that more than half of nurses (54.70%) took on additional employment, and the remaining 45.30% worked in 1 place of work. Women dominated in both groups: 85.94% among those working several jobs and 97.17% in 1 facility. The study by Malinowska-Lipień et al. showed that 44% of nurses out of 1023 surveyed, who were registered with the Małopolska District Chamber of Nurses and Midwives, took on additional employment [12]. The study by Stephenson [20], which was conducted among readers of online medical journals in Great Britain, shows that out of 800 respondents, 47% declared additional cooperation with nurse banks and/or a nurse agency. Therefore, there is a tendency to take up additional employment of nurses not only in Poland but also in European countries.

The main reason for additional employment is financial issues, which motivate 82.81% of respondents, while 78.91% would resign from it if the main job provided adequate earnings. Other reasons, such as the desire to use free time, are rare. Unfortunately, additional work is associated with a higher level of burnout and EE due to the greater burden. Studies by Ogińska and Żuralska [21], as well as Kowalczuk et al. [22] have shown that low nurses' salaries significantly affect the development and emergence of burnout. In 2022, Poland introduced the “Act on the method of determining the minimum basic salary of certain employees employed in healthcare facilities” [23], which aimed to increase nurses' salaries by introducing pay rises depending on the level of education in individual professional groups. However, some hospitals do not pay salaries in accordance with the provisions of the Act, because it refers to the required qualifications, not the ones they have. As a result, many nurses decide to take additional employment to compensate for the differences in earnings. According to the report of the Organisation for Economic Co-operation and Development (OECD) [24], before the COVID-19 pandemic, an upward trend in nurses' salaries was observed in many countries of Central and Eastern Europe (Hungary, Poland, the Czech Republic, Slovakia). However, taking US dollars as the currency, the data from 2021 show that Polish nurses earned an average of USD 45 000 per year, which is USD 7000 less per year than the average of the member states [24].

Nurses who took on additional work felt its negative impact on their mental health and a sense of fatigue. Studies conducted in Hungary have shown a relationship between the level of mental strain and the risk of developing burnout symptoms [25]. In the authors' own study, frequent fatigue in the last 6 months was reported by 87.50% (N = 112) of multiworkers, compared to 74.53% (N = 79) of monoworkers. Respondents working in multiple places more often reported symptoms such as sleep disorders, muscle and joint pain compared to those working in 1 place. In the study by Khamisa et al. [26], EE and DE were shown to be associated with anxiety, insomnia and headaches. It has also been proved that poor staff management and inadequate equipment significantly contribute to the occurrence of burnout. Additionally, the study by Khatatbeh et al. [27]. described the impact of shift work and night shifts and related sleep problems on the development of burnout compared to nurses working regular day shifts. Workload remains a key factor negatively affecting the health and quality of life of nurses.

The study by Nowakowska and Rasińska [28] conducted on 405 nurses revealed that short work experience promotes personal burnout, which may result from the lack of experience in coping with occupational stress. In turn, long-term work experience in 1 position is associated with burnout resulting from routine and monotony. Nurses from conservative wards were more susceptible to burnout due to the high level of responsibility and stress. The study by Wieder-Huszla et al. [29] showed that a high level of professional burnout is observed in nurses working in long-term and palliative care facilities. Researchers emphasize the need for individualized support for nurses, especially younger ones and those working in demanding conditions. The proposed solutions include stress management training, psychological support, professional development opportunities and job rotation, which can reduce routine and increase employee well-being [29]. The authors' own research shows that longer work experience may lead to greater EE, but also to a greater sense of professional success. The study by Kędra et al. [30] confirmed that older nurses more often experience a sense of burnout, but they show less knowledge about this phenomenon [30]. In the conducted authors' own study, more than one third of people working in an additional job (35.16%) has 5–20 years of work experience. Similar results were obtained in the study by Dąbrowska-Chołostiakow et al. [31], where additional employment was undertaken by nurses with 10–30 years of work experience. Higher levels of experience among nurses are associated with a higher incidence of burnout, and employment in an additional job may further increase the level of burnout.

The study on the impact of nurses working multiple jobs on their burnout and fatigue is an innovative approach to analysing the problems that healthcare works have to face. Although it is crucial for assessing the quality of care and well-being of staff, it has rarely been analysed in detail in the scientific literature so far. This indicates the need for further research in this area to understand the mechanisms leading to burnout and fatigue. The need for systematic actions, such as salary increases, improved working conditions and support in coping with stress, was also emphasized, which will contribute to improving the quality of life of nurses and the efficiency of the healthcare system.

One of the significant limitations of the study is the data collection method used. The use of an online survey may have limited access to some respondents, especially those who do not have access to the Internet or do not actively use digital technologies. Another limitation is the lack of consideration of other variables that may significantly affect burnout and fatigue. Factors such as working conditions, number of working hours, availability of social support, family situation or lifestyle were not included in the analysis, which may lead to a simplified interpretation of the results. It is also worth noting that the study focuses mainly on the negative aspects of double employment, such as fatigue and mental health, while omitting potential benefits such as professional development, increased competence or greater financial satisfaction. This onesided nature of the analysis may limit full understanding of the phenomenon. In subsequent studies, it is planned to expand the research group both in terms of numbers and professional diversity, including other medical workers taking on additional employment. It will also be important to consider a broader spectrum of factors, both negative and positive, that may affect burnout and fatigue, as well as the potential benefits of double employment. This approach will allow for a more comprehensive picture of the problem being analysed and will contribute to deepening knowledge in this area.

## CONCLUSIONS

The financial factors are the main reason for nurses taking on additional employment. Therefore, ensuring adequate compensation within a single full-time position should be a priority, in order to reduce the need for multiple job holdings. The analysis of the results showed that working in >1 place significantly increases the level of fatigue compared to employment in 1 facility. Nurses employed in >1 full-time position were characterized by higher levels of burnout and increased EE. Over 87% of respondents who worked additionally also regularly felt tired. In addition, these people experienced fatigue and headaches more often. Higher levels of fatigue correlated with more intense burnout and greater EE. Healthcare institutions should implement systems to monitor workload and fatigue levels among nursing staff, particularly for those employed in >1 facility. Given the significant association between older age and increased EE, it is advisable to introduce support mechanisms for senior nurses, such as workload reduction or access to psychological support. Although other demographic and professional variables did not show a statistically significant impact on occupational burnout, further research and the implementation of evidence-based systemic changes are recommended to support the health and well-being of the nursing workforce.
